# W. A. Townsend

**Published:** 1876-04

**Authors:** 


*W. A. Townsend, Publisher and Bookseller,* New York, requests us to say, that M. C. Lockwood is not authorized to use his name in procuring subscribers for various medical journals. His engagement confines him exclusively to Braithwait's Retrospect.

